# Anthropometrics of Estonian children in relation to family disruption: Thrifty phenotype and Trivers–Willard effects

**DOI:** 10.1093/emph/eoab022

**Published:** 2021-07-20

**Authors:** Markus Valge, Richard Meitern, Peeter Hõrak

**Affiliations:** Department of Zoology, University of Tartu, Vanemuise 46, Tartu 51014, Estonia

## Abstract

**Background and objectives:**

The thrifty phenotype hypothesis proposes that at resource limitation, the growth of some organs/tissues is selectively spared to preserve more critical ones, such as the brain or lungs. The Trivers–Willard hypothesis (TWH) predicts that boys are more vulnerable in the case of resource limitation than girls. Both hypotheses were tested in children from disrupted families, differing in the extent of deprivation/adversities imposed on them.

**Methodology:**

In a retrospective cohort study in the mid-20th century Estonia (Juhan Aul’s database), different types of orphans and children of divorced parents (treatment groups; *n* = 106–1401) were compared with children from bi-parental families (control groups; *n* = 2548–8648) so that children from treatment groups were matched with control children on the basis of sex, age, year of birth, urban versus rural origin and socioeconomic position.

**Results:**

Children in orphanages suffered strong growth suppression, best explained by psychosocial deprivation. Their feet were on average 0.5 SD shorter than the feet of the controls, followed by height, leg/torso ratio and cranial volume that differed from controls by ca 0.4 SD. Weight difference was 0.2 SD units, while body mass index did not differ from controls. The growth of boys and girls in orphanages was suppressed to the same extent. Boys whose mothers were dead were relatively smaller and less masculine than girls from such families. Fathers’ absence was unrelated to growth suppression. Sons of divorced parents had broader shoulders than boys whose fathers were dead.

**Conclusions and implications:**

Prediction of TWH about the greater vulnerability of male growth may hold under some conditions but not universally. Predictions of the thrifty phenotype hypothesis were partly supported: trunk growth was spared at the expense of leg growth; however, no evidence for brain sparing was found. Comparison of children of divorced versus dead fathers may appear useful for indirect assessment of sexual selection on offspring quality.

**Lay Summary:** Boys and girls in orphanages suffered similarly strong growth suppression, best explained by psychosocial deprivation. Boys whose mothers were dead were relatively smaller and less masculine than girls from such families. The occurrence of sex-specific associations between family structure and children’s growth depends on the type of family disruption.

## INTRODUCTION

When the growing organism is short in resources, it faces a trade-off of allocating these limited resources optimally between competing organs and tissues (reviewed by [1]). According to the thrifty phenotype hypothesis [2, 3], reduced energy supply affects tissues of developing organisms to different degrees. Extensions to this hypothesis have proposed that at resource limitation, essential organs such as the brain, heart and lungs are protected at a cost to other tissues/body parts such as limb length and skeletal muscle (reviewed by [4, 5]). These hypotheses are consistent with findings that childhood leg growth is the component of linear growth most sensitive to nutrition [5–10]. The Trivers–Willard hypothesis (TWH) predicts that boys are generally more vulnerable to developmental stress than girls [11, 12] because at resource limitation during growth, selection will favour production and investment in daughters, so long as daughters are likely to be mated, whereas sons in poor condition are likely to be out-competed by other males [13]. However, the question of whether the trade-offs between investment into the growth of different body parts also differ between sexes is still unclear [1].

Resource limitations experienced by growing children vary in severity and nature. Perhaps the most extreme case in contemporary societies is psychosocial growth failure, universally reported in institutionalized children [14]. In such case, severe suppression of height, weight and head size occurs as a result of nutritional insufficiencies as well as suppression of the growth hormone/insulin-like growth factor (GH/IGF-1) axis, caused by social deprivation [14, 15]. An extensive meta-analysis [16] established that international adoption of institutionalized children leads to substantial catch-up growth of height and weight but not of head circumference, demonstrating differential plasticity of growth of different organs.

The growth of children may also be stunted in the general population in modern societies if they have many siblings or overcrowded households [17, 18], and/or low family income [18, 19] or absent genetic father [18, 20]. Again, head/brain size seems to be particularly sensitive to non-material parental investment, as children experiencing harsh parenting [21], childhood adversities (parental separation, abuse, violence etc) [22], insensitive parental care in infancy [23] or living with step-father [24] develop smaller brains or skulls. Yet, the question about the relative importance of different adversities on the growth of different body parts stays poorly understood as, for instance, studies that have concentrated on brain growth and development have seldom measured other anthropometric traits and *vice versa*.

This study maps the associations between family structure and a diverse set of anthropometric traits of Estonian children in the middle of the 20th century. Children growing up in orphanages, those with dead mothers or fathers, and those with divorced fathers (i.e. fathers that are absent due to divorce) are compared with control children in bi-parental families. These growth environments are expected to differ in the extent of deprivation/adversities imposed on children.

### Predictions

Based on previous reports (e.g. [15]), we predicted that children from orphanages would display the most severe growth stunting (particularly in head size) compared with children from all other family types. Because mothers invest substantially more time and effort in parenting as compared with fathers [25] and maternal orphanhood affects children more strongly than paternal one [26], we predicted that children whose mothers were dead would appear the second hardest disadvantaged category (after double orphans) in terms of linear growth. Given the higher share of mothers in parenting and the importance of social stimulation for brain growth, we also predicted that maternal orphans have smaller crania than children from bi-parental families.

As regards fatherless children, we considered three hypotheses. First, assuming that divorced/separated fathers were obliged to pay alimony, one might predict that their children were better off than children of dead fathers in terms of material resource availability, and hence, suffered fewer resource limitations on growth as compared with children from bi-parental families or children of dead fathers. Alternatively, men prone to divorce might be genetically different from the rest of the population concerning their body size and shape. Genetic risk factors partly influence the probability of divorce, and a high genetic correlation exists between the liability to divorce and risk for major depression [27], which, in turn, is genetically linked with short stature [28]. On the other hand, men prone to divorce are high in testosterone [29–31]; thus one might expect that their children possess more masculine bodies than controls.

Additionally, it is possible that family/marital conflict that precedes divorce/separation has adverse effects on children’s wellbeing and growth via direct non-genetic pathways [32, 33]. Under both genetic and non-genetic pathways of divorce-stress-induced growth suppression, we would predict that children of divorced fathers are smaller than those from bi-parental families and also smaller than children whose fathers were dead (assuming that fathers’ death was random concerning the genetic component of their body size; see [34]). Alternatively, if divorced fathers possess heritably high testosterone levels, we predict that their children have a more masculine body build than children whose fathers were dead or living with their families.

### Anthropometric traits

Altogether we examined 14 anthropometric traits. Of these, weight and body mass index (BMI) can be considered as indices of nutritional state. Absolute and relative leg length (leg length/sitting height) are markers of pre-pubertal growth conditions, whereas trunk (which houses major organs whose function may be impaired by poor growth) obtains its final dimensions after childhood [10]. Thorax circumference is a correlate of respiratory capacity in children [35]. Hip width develops under the influence of ovarian steroids and age when hips attain adult dimensions strongly correlate with menarcheal age (Ellison 2001), so this measure can be considered a proxy of the rate of sexual maturation in girls. Cranial volume is sensitive to nutrient limitations [36] and parental stimulation [23] during growth and it correlates both phenotypically [37, 38] and genetically [39] with cognitive abilities and educational attainment. In our study population, cranial volume and height predict educational attainment independently of socioeconomic background [40]. Our dataset also includes several testosterone-dependent traits such as face width and roundness, shoulder width and shoulder/hip ratio and handgrip strength. Grip strength is also recognized as a marker of physical ability and vigour which is predictive of short- and long-term morbidity and mortality in adult populations (e.g. [41]).

### Exact covariate matching

Our method of comparing different types of orphans and children of divorced fathers (treatment groups) with children from bi-parental families (control groups) is based on the exact covariate matching. Each child from each treatment group is matched with children from bi-parental families based on sex, age, year of birth, urban versus rural origin and family socioeconomic position (SEP). The general idea behind matching is to approximate the outcome of a randomized experiment for an observational study, and thereby reducing potential selection bias and increasing the precision and efficiency of the estimates [42]. Associations between the disrupted family status of children and their anthropometric traits is tested in linear models where each individual in the control group is assigned a weight based on the ratio of treated to control individuals within each set [43]. To test whether boys are more vulnerable to resource limitation than girls, models for the effects of treatment on anthropometric traits are tested for the sex*treatment interaction.

## METHODS

Data on morphometric measurements and family background were obtained from the anthropometric study performed between 1956 and 1969 (for the historical background of this sample see [44, 45]). The aim of the original project, initiated by the founder of physiological anthropology in Estonia, Prof Juhan Aul (1897–1994), was typical to physical anthropology of the first half of the 20th century: to map the diversity of body size and shape (somatotypes), as well as rates of growth and pubertal maturation of Estonian children concerning their birth years, geographic location, social and urban versus rural origin. He also meant that knowledge of anthropology is applicable and could be used in the advancement of public health purposes, including nutrition and health care, in physical education, in the production of clothing, footwear, furniture and other essentials, as well as in education, vehicle construction etc. [46].

The current dataset covers Estonian schools in major cities (Tallinn, Tartu, Pärnu, Kohtla-Järve) and extensive rural areas in mainland Estonia (Supplementary Fig. S1). Since Russian-language schools are missing from the sample, the study is nationally representative for the Estonian-speaking population only. Children were measured once but ca 60% of them could be later identified in the Population Registry, enabling assessment of their education and life-history parameters [40, 45, 47].

Details for measurements are described in [45]; the leg/torso ratio denotes leg length ratio to sitting height. Age- and sex-specific residuals of anthropometric traits were calculated from generalized additive models in which the focal trait was regressed against smooth nonparametric functions of age (in days) and birth date using package ‘gam’ for R [48] ((R syntax: focal_trait ∼ s(age) + s(birth_date)). We included birth date as a predictor to account for the steady increase in age-adjusted body dimensions over the study period (see [44]). Residuals were then standardized to z-scores within sexes. All results presented here are based on these standardized residuals rather than raw trait values. The full dataset for calculation of residuals of morphometric traits consisted of 12 465–15 253 girls (depending on the trait) and 9791–11 842 boys (age range 6.4–20.0 years, mean = 12.7 ± 6.4 (SD) years, born between 1936 and 1962).

From this sample, we identified four subsets of different types of children from disrupted families who were matched with control children living with both birth-parents based on sex, age, year of birth, urban versus rural origin and SEP. SEP (highest in the family) was assessed on the basis of parental professions recorded during data collection and assigned into three categories: unskilled manual workers, skilled manual workers or non-manual workers. For all matching calculations, we used the exact matching procedure with R package MatchIt [43], allowing maximally 1-year difference in age between treatment and control observations. Matching is an alternative to regression analysis to identify the impact of a treatment variable on an outcome variable that allows for more flexibility relative to regression and weighting of observations. In many cases, one can assume that the careful matching methods will result, relative to a standard regression model, in a more precise and efficient estimate of the coefficient of interest [42].

First of our subsets consisted of 266 children residing in orphanages who were matched against 5616 control children. Differently from all other matchings, SEP could not be used for matching of children from orphanages. The second subset consisted of 371 children who had reported that their mothers were dead; these were matched against 6710 controls. Third subset consisted of 2401 children who reported that their fathers were dead; these were matched against 15 471 controls. Fourth subset consisted of 842 children who reported that their fathers were divorced; these were matched against 8786 controls. The high number of children with dead fathers enabled us to construct also the fifth dataset where 591 children with divorced fathers were matched against 984 children with dead fathers as controls. Descriptive data on all these subsets are presented in Supplementary Table S1. We had no information on whether the children who reported that one of their parents was dead or divorced had a step-parent in their family. Given the highly female-biased sex ratio characteristic to the post-war situation, we consider it likely that children who reported that their mother was dead had a high probability of living with step-mother, whereas children with dead or divorced fathers were less likely to have a step-father.

Associations between disrupted family status of children and their anthropometric traits is tested in linear models where each matched control unit has weight proportional to the number of treatment units to which it was matched, and the sum of the control weights is equal to the number of uniquely matched control units. First, we ran linear models with treatment as a factor for boys and girls separately (R syntax: lm (formula = focal_trait ∼ treatment, data = data, weights = weights)). Thus, regression coefficients equal the differences between mean trait values of children in control and treatment groups in SD units. To test for the sex differences of treatment effects on anthropometric traits, we ran another set of models including sex*treatment interactions (ANOVA (Analysis of Variance) function in R package car; R syntax: lm(formula = focal_trait ∼ treatment + sex: treatment, data = data, weights = weights)). R code for matching is presented in Supplementary Material.

All the linear measures of size recorded in our study are positively correlated with each other and also with grip strength [40, 45]. Confidence intervals that do not include one are considered statistically significant at the 95% level. Sample sizes vary slightly between analyses because participants differed with respect to the number of anthropometric traits recorded. Data processing was performed anonymously under the licence of the Research Ethics Committee of the University of Tartu (protocol no. 275/T-1, issued on 20.11.1017) and approved by the Estonian Data Protection Directorate (Decision n2 2.2.-1/17/55, issued on 30 January 2018).

## RESULTS

**Figure 1.**
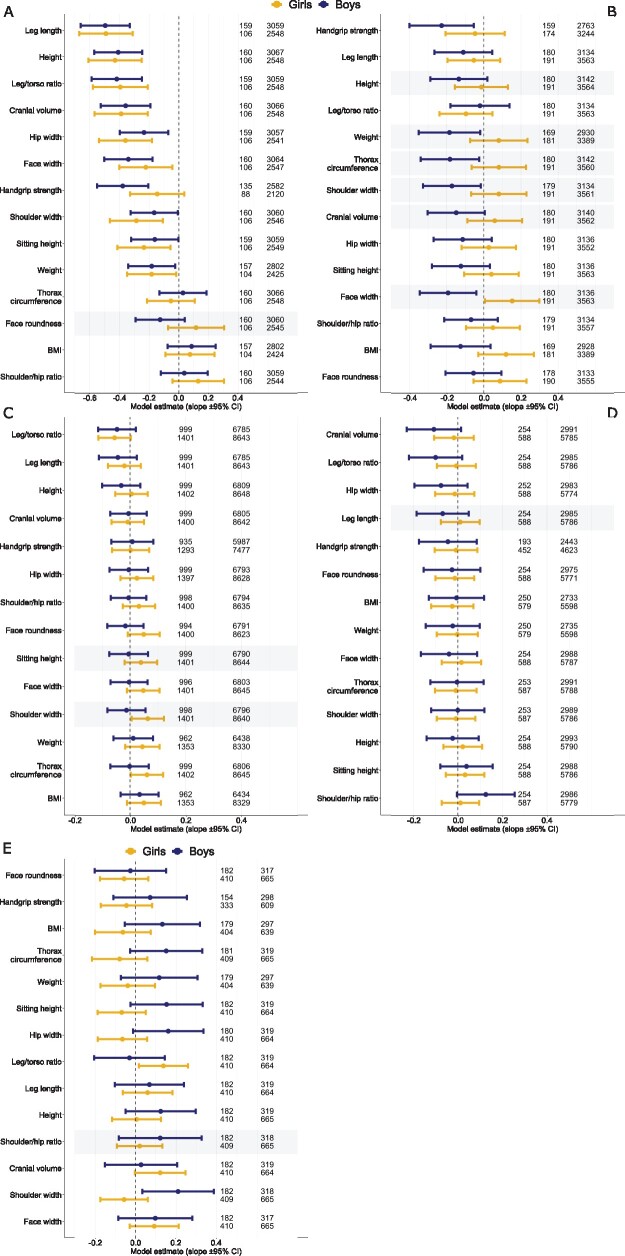
Comparison of anthropometric traits in disrupted versus bi-parental families. Regression coefficients equal the differences between mean trait values of children in control and treatment groups in SD units in linear models. Sample sizes for treatment and control groups are indicated at right. Shaded areas denote significant trait*treatment interaction terms. F-statistics and p-values are given in Table S1 in ESM1. (**A**) Children from orphanages versus controls. (**B**) Children with dead mothers versus controls. (**C**) Children with dead fathers versus controls. (**D**) Children with divorced fathers versus controls. (**E**) children with divorced fathers versus children with divorced fathers

Children from orphanages differed from bi-parental control children for eight of 14 anthropometric traits. The most sensitive trait was leg length (difference from controls = 0.5 (95% CI = 0.7–0.3) SD units; *P* < 0.00001), followed by height and height/torso ratio and cranial volume (difference from controls = 0.4 (95% CI = 0.6–0.2) SD units; *P* < 0.00002; Fig. 1A and Supplementary Table S1A). Orphans also had narrower hips, faces and shoulders, shorter sitting heights and lower weights (difference from controls = 0.2–0.3 (95% CI = 0.5–0.0) SD units; *P* = 0.00004–0.046), boys from orphanages were also weaker (difference from controls = 0.5 (95% CI = 0.6–0.2) SD units; *P* < 0.00002), whereas girls from orphanages did not differ from controls for grip strength (*P*-value for sex*trait interaction, however, was only 0.062). The *P*-value for sex*trait interaction was 0.026 in case of face roundness, suggesting that orphan status was associated with relatively narrower faces in boys and relatively wider faces in girls (although when analysed separately by sex, no differences from controls emerged; Fig. 1A and Supplementary Table S1A). Thorax circumference, BMI and shoulder/hip ratio were not associated with orphan status.

Girls whose mothers were dead differed from controls only by wider faces (difference = 0.15 (95% CI = 0.01–0.30) SD units; *P* = 0.041). Boys, in turn, had narrower faces than controls (difference = 0.19 (95% CI = 0.35–0.04) SD units; *P* = 0.013). Additionally, sons of dead mothers were weaker and lighter and had narrower shoulders and smaller thorax circumferences than controls (all differences in the magnitude of about 0.2 SD (95% CI = 0.4–0.02); *P* = 0.010–0.029; Fig. 1B and Supplementary Table S1B). Inspection of sex*trait interaction terms suggests that maternal orphanhood had sex-specific effects on 6 traits of 14: height, weight, thorax circumference, shoulder width, cranial volume and face width. In all cases, the effects were in the same direction: boys were more negatively affected than girls.

Having a dead father was not associated with boys‘ traits, whereas girls with dead fathers had slightly wider shoulders and greater thorax circumferences than controls (difference = 0.06 (95% CI = 0.00–0.12) SD units; *P* = 0.032–0.040; Fig. 1C and Supplementary Table S1C). Shoulder width also displayed clear interaction with sex (*P* = 0.008), indicating that paternal orphanhood was associated with wider shoulders in girls and narrower shoulders in boys. Sitting height revealed similar (although weaker) interaction (*P* = 0.030).

Children of divorced fathers did not differ from controls regarding any trait (Fig. 1D and Supplementary Table S1D). We found weak (*P* = 0.037) evidence for the sex*leg length interaction, suggesting that leg length of boys was more strongly associated with divorce than that of girls. When compared with children with dead fathers, daughters from divorced families had higher leg/torso ratios (difference = 0.14 (95% CI = 0.02–0.26) SD units; *P* = 0.027) and boys had broader shoulders (difference = 0.21 (95% CI = 0.03–0.39) SD units; *P* = 0.020). Figure 1E and Supplementary Table S1E). Sex*shoulder width interaction (*P* = 0.025) suggests that compared with daughters, sons of divorced fathers had more masculine body shape than sons of dead fathers.

In order to test whether the 1-year age difference between treatment and control samples could affect our results, we ran a sensitivity analysis with a half-year maximum age difference for matching. All the effects were in the same direction and almost indistinguishable from the results obtained with the full dataset (Supplementary Fig. S2 and Table S3).

## DISCUSSION

### Psychosocial growth retardation

Our study revealed several robust patterns in associations between family structure and body size/shape of children that are relevant for understanding the possible trade-offs in resource allocation between different body parts under resource limitation. Children from orphanages displayed the most extreme differences compared with control children in bi-parental families: their feet were on average 0.5 SD shorter than the feet of the controls, followed by height, leg/torso ratio and cranial volume that differed from controls by ca 0.4 SD. The weight difference was 0.2 SD units, whereas BMI did not differ between children from orphanages and controls. These findings, particularly as regards height and cranial volume, are consistent with the results of the studies of institutionalized children worldwide [15, 16].

A relatively weak association between orphan status and weight and the absence of the association between orphan status and BMI suggests that children residing in orphanages were not calorie deficient. Thus, it is likely that the small body and head dimensions of children in orphanages must result from some mechanism other than low calorie intake, such as deficits of specific micronutrients like iron [14] For instance, in a sample of Estonian children measured at the end of the past century, self-reported meat shortage was associated with below-average cranial volume [24]. One should also not underestimate the adverse effects of psychological stress on growth that are long known in laboratory animal models as well as humans. For instance, in psychosocially short stature, linear growth delay, especially in children with normal weight, may reflect the effects of chronic stress on the GH system. Under such a scenario, elevated levels of corticotropin-releasing hormone and cortisol under conditions of psychosocial deprivation are believed to mediate inhibition of GH/IGF-1-system, leading to suppression of linear growth (reviewed by [14, 49]). Our finding of small cranial volumes of children residing in orphanages is well consistent with substantial experimental and clinical evidence about the role of psychological stress (via GH/IGF-1 pathway) in the determination of brain development and growth (reviewed by [50]) that eventually limits head size [51].

On the other hand, despite the well-known effects of altered hypothalamic-pituitary-adrenocortical (HPA) activity on post-natal growth described above, one should also not ignore the fact that parents of institutionalized children do not comprise a random sample of the population concerning alcohol abuse, psychiatric disabilities and poverty/misery [52]. For instance, prenatal alcohol exposure is common among institutionalized children in Eastern Europe [53] and is associated with retardation of height and cranial volume that may persist into adulthood [54]. Because we lack the information about the parents of institutionalized children, we cannot distinguish to what extent their small size was caused by psychosomatic growth suppression versus prenatal and hereditary effects. We consider it likely that parents of children in orphanages in our study comprised a mixture of those whose children were institutionalized due to their inability to perform normal parenting, and those whose children were institutionalized because the parents were dead or separated from children due to war or Stalinist repressions (see [44]). However, the proportion of either types of parents remains unknown.

Our findings from orphanages offer partial support for the hypothesis that at resource limitation, essential organs such as the brain, heart and lungs are protected at a cost to other tissues/body parts such as limb length and skeletal muscle [4, 5]. Legs of institutionalized children were on average 0.5 SD shorter than the legs of controls, whereas the difference in sitting height was only about 0.2 SD and thorax circumference was not different from that of controls. In particular, the finding of ‘sparing‘ of thorax circumference supports the version of the thrifty phenotype hypothesis emphasizing the need for preserved lung function in order to support brain energy metabolism [7]. On the other hand, the effect of orphan status on cranial volume was rather prominent (ca 0.4 SD) and in the same magnitude as the effects on height and leg/torso ratio. Preferential protection of brain growth was thus not observed. Further, extensive meta-analyses have shown that brain growth and function of institutionalized children are more strongly suppressed than height and show incomplete catch-up after adoption from orphanages to foster homes [15, 16].

On the other hand, the contradiction with the brain-sparing concept may be only apparent, considering that absence of parental, and particularly maternal, care is likely to be an evolutionarily novel situation. For instance, it may be possible that without the assistance of mother or kin, infants’ chances for surviving in our ancestral environment were negligible. In such a case, psychosocial suppression of head growth appears as an evolutionarily novel phenomenon that does not require adaptive explanation. Further, even though brain growth is sensitive to psychological stress also in general population [21], a sizable proportion of children in orphanages may possess parents with small brains, so that the average cranial volume of institutionalized children is small for hereditary reasons. Such scenario would explain why brains of some but not all children show compensatory growth and development after adoption from orphanages (see [54]). Finally, the observed pattern of absence (or incomplete) brain sparing of institutionalized children would also be compatible with the ‘adaptive mental retardation hypothesis‘ by Reser [55]. The hypothesis states that suppressed brain growth represents a predictive-adaptive response to environmental cues that communicate to the foetus that, because it will be neglected of maternal investment, developing a metabolically saving brain will be an effective ecological strategy that enables maintaining of reproductive function (and still permits settling in a less skill-intensive niche).

### Sex differences in growth responses to parental loss

According to the TWH, the reproductive success of male mammals is more sensitive to their physiological condition (vigour, size) than that of females. Hence, at resource limitation during growth, selection will favour production and investment in daughters, so long as daughters are likely to be mated, whereas sons in poor condition are likely to be out-competed by other males [13]. The TWH suggests that selection has shaped mechanisms to direct parental investment toward the offspring most likely to increase their fitness. The prediction of TWH that mothers in good condition should bias their progeny sex ratios towards sons and mothers in poor condition towards daughters has gained quite good support in non-human mammals and few human studies (reviewed by [56, 57]). On the other hand, the predictions of sex-specific parental nurturing in post-natal life in humans (a category into which this falls) have yielded inconsistent results [56–58].

An epidemiological extension of the TWH [11] posits that the rationale mentioned above of sex-specific investment into offspring explains greater male vulnerability in response to environmental stress in early life. According to Wells [11], natural selection is predicted to have favoured increased male vulnerability to any given degree of early environmental stress, either an infectious disease, injury or malnutrition. Our findings cast doubt on the general validity of this prediction as the growth of the boys and girls in orphanages was suppressed to the same extent, and none of the measures of body size showed sex-specific vulnerability. Similarly to our study, two meta-analyses found no sex difference in the growth of institutionalized children [15, 16]. However, in the latter study, boys had more cognitive and socioemotional development delays, and girls had more delays in physical health.

In contrast to the absence of sex-specific growth suppression of children from orphanages, children whose mothers were dead showed a clearly distinct pattern. Girls whose mothers were dead did not show any signs of growth suppression, whereas boys were weaker and lighter, had smaller thorax circumference and narrower shoulders and faces than boys from the bi-parental families (Fig. 1B). The magnitude of growth suppression was, however, considerably smaller than in the case of children from orphanages. Inspection of sex*trait interaction terms indicates that maternal orphanhood had sex-specific associations with height, weight, thorax circumference, shoulder width, cranial volume and face width. Such patterns are consistent with the prediction of TWH that environmental stress affects boys more strongly than girls. Three traits that showed sex-specific associations with maternal orphanhood are markers of past and/or present testosterone exposure—grip strength, shoulder width and face width (see [40, 45, 59, 60]). Thus, among the children whose mothers were dead, boys appeared less masculine than controls and girls appeared more masculine than boys, and concerning face width, also more masculine than controls. Other sex-specific effects of parental loss on testosterone-dependent traits emerged in children from orphanages (face roundness, Fig. 1A) and in children whose fathers were dead (shoulder width and thorax circumference, Fig. 1C).

High prevalence of sex*trait interactions among children with dead mothers would suggest that growth of (particularly testosterone-dependent) traits is more sensitive to maternal absence in boys than in girls. However, the published evidence in support of such claim is controversial. Sex hormones are regulated by the hypothalamic-pituitary-gonadal (HPG) axis and, given that the HPA and HPG axes share similar neuroendocrine pathways, there is crosstalk between both pathways, so that in adult humans and animal studies increased activation of the HPA axis inhibits the HPG axis and *vice versa* (reviewed by [61]; but see [62]). However, studies in adolescents have shown the opposite patterns, i.e. the positive coupling of HPA and HPG axes [61, 63]. Evidence about sex differences in stress responsiveness is unequivocal as well [62, 64]. However, assuming that association between maternal death and suppression of growth of testosterone-dependent traits in boys reflect slower pubertal development, our results are comparable with these of Sheppard et al. [32], showing in a large US sample that boys (but not girls) whose mother or father had died before age seven were shorter than controls and that their later age of puberty fully mediated this association.

Findings that boys whose mothers were dead appeared relatively smaller than girls do not appear typical in bereavement studies. A recent meta-analysis found that adolescent females reported higher levels of internalized grief responses (*d* = 0.18, six studies) and higher levels of post-traumatic stress disorder symptoms (*d* = 0.36, four studies) in response to the death of parent or sibling [65]; see also [66]. Yet, other studies have found no sex differences in psychological sequelae of parental death [27, 67, 68] or indicated that girls are better equipped to deal with stressful life events, such as the death of a mother [69, 70].

### Fathers’ absence

Due to the high number of children whose fathers were dead (2401 vs 15 428 controls), we had the highest test power for detecting the effects of paternal death on children’s growth (compared with other categories of familial disruption). Yet, we did not find any associations between fathers’ death and suppressed growth. This finding differs from the study of 16 207 US children interviewed between 1938 and 1962 by Alfred Kinsey, who showed that paternal death before age seven was associated with shorter stature in boys [32]. It is possible that discrepancy between the findings of these studies results from different reasons of premature paternal deaths. Although the proportion of children with dead fathers was rather similar (9.5% of the total sample in the Kinsey’s study and 10% in the whole Aul’s dataset), one might expect that proportion of war-associated deaths was smaller in the US sample than in Estonia that suffered disproportionate population loss due to WW II and Stalinist terror (see [44]). Also, the median year of birth of participants in Kinsey’s study (1920) was 25 years earlier than in our sample involving dead fathers (1945; Supplementary Table S2), which might further explain the greater sensitivity of children in Kinsey’s sample to external adversities. For instance, the availability of antibiotics and new vaccines since the middle of the 20th century correlated with children‘s growth in Aul’s dataset [44].

Similarly to fathers’ death, paternal divorce/separation was not associated with growth suppression as compared with bi-parental controls (Fig. 1D). However, when children of divorced fathers were compared with those whose fathers were dead, it appeared that sons of divorced fathers had wider shoulders than boys whose fathers were dead (0.2 SD difference; Fig. 1E). This finding offers some indirect support to the hypothesis that, in contrast to boys whose fathers died at a relatively young age, sons of men liable to the divorce may inherit a masculine body build from their fathers. Such an explanation would be consistent with findings that men prone to divorce have high levels of circulating testosterone [29–31] and genetically high androgenicity (shorter polyglutamine tandem repeats (CAGn) within the androgen receptor [AR] gene [71]). It should be noted, however, that CAGn polymorphism cannot be a mechanism for father–son inheritance of masculine body build because AR gene is located on the x chromosome in humans; thus, under such scenario we would have rather observed that daughters of divorced fathers are characterized by highly masculine body build. This, however, does not contradict the high heritabilities of testosterone-dependent traits (e.g. h^2^ for shoulder width was 0.78 for Chinese boys and 0.56 for girls, respectively [72]; for Bengalese children aged from 6 to 19 years, h^2^ for shoulder width varied between 0.30 and 1 [73]).

Our finding that sons of divorced fathers had wider shoulders than boys whose fathers were dead is consistent with ‘sexy son‘/’good genes’ hypotheses [74, 75]. According to these, masculinity and attractiveness are presumed to underlie male allure, and the commonality in father’s and son’s appearances depends on shared genes controlling androgenic response or genes influencing body growth [76]. Previous studies have found that not all testosterone-dependent traits are similarly attractive. A recent meta-analysis showed that strength/muscularity was the strongest and only consistent predictor of both mating and reproduction in men, whereas facial masculinity did not significantly predict either [77]. Such associations are likely highly context-dependent; for instance, our study failed to replicate findings of Lauringson *et al*. [24] that children living with step-father had smaller crania than children in bi-parental families. Yet, our results suggest that comparison of children of divorced versus dead parents may appear a useful model for indirect assessment of the sexual selection on offspring quality.

### Limitations, conclusions and implications

The limitation of this study is that we had no information on whether the children who reported that one of their parents was dead or divorced had a step-parent in their family. We thus could not control for the possible effects of step-parents on family welfare. The study protocol of Juhan Aul did not contain a questionnaire that had enabled detailed assessment of the extent of deprivation in families. Also, we lacked information about children’s age when parental death or divorce occurred.

Our study design did not account for the family size because this would have excluded the possibility of using exact matching procedure: in such a case, we would have run in problems of finding sufficient number of treatment-control matches. However, we don’t think that ignoring sibling number would have affected our results much because previous study in our population showed that the effect of sibling number on growth is very small [44]. Also, we had no information about the extent of child work but we believe that this factor is much covered with the urban/rural factor in matching. Specifically, rural children in general were highly likely to participate in field labour in garden plots owned by the entire rural population and also in summer work in collective farms (kolkhozes) [44]. We believe that most of the effects of disease environment/infection on growth are covered by matching for birth year as there was a clear decline in infant mortality during the study period [44].

The main finding of this study is that different types of family disruption were associated with different growth suppression patterns. Children in orphanages suffered strong linear growth suppression, which can be best explained by psychosocial deprivation. At that, the growth of boys and girls was suppressed to the same extent. Thus, our study (along with previous findings of institutionalized children) provides evidence that predictions derived from the TWH about greater male vulnerability in response to environmental stress in early life do not hold universally. Data on children from orphanages supports the prediction of the thrifty phenotype hypothesis that trunk growth is spared at the expense of leg growth in response to environmental stress. However, no evidence for brain sparing was found.

In contrast to children from orphanages, children whose mothers were dead showed several sex differences in growth compared with children in bi-parental families. In particular, boys appeared relatively smaller and less masculine than controls, whereas girls appeared relatively more masculine than boys. This suggests that stress associated with mothers’ absence may delay puberty in boys. Fathers’ absence, either due to divorce or death, was not related to growth suppression. Sons of divorced fathers had broader shoulders than boys whose fathers were dead, consistent with ‘sexy son‘/’good genes’ hypotheses. This finding suggests that comparison of children of divorced dead parents may appear as useful model for indirect assessment of sexual selection on offspring quality. As for practical implications for public health, our study reinforces the importance of leg length as the most sensitive anthropometric marker of childhood growth conditions that deserves attention in monitoring institutionalized children’s growth.

## Supplementary data

Supplementary data is available at *EMPH* online.

## Availability of data and materials

Commented R code for matching procedures is given in the Supplementary Material S1. Original data are available in Supplementary Material S2.

## Supplementary Material

eoab022_Supplementary_Data
