# Supplementary material for: Anthropometrics of Estonian children in relation to family disruption: Thrifty phenotype and Trivers–Willard effects
Source: Evol Med Public Health. 2021 Jul 20;9(1):276–86. doi: 10.1093/emph/eoab022 (PMC8445393; doi:10.1093/emph/eoab022)
Supplement: eoab022_Supplementary_Data [file eoab022_supplementary_data.zip › ESM1.pdf]

Table S1A. Effects of orphan status on anthropometric traits of children (separately for boys and girls; R syntax: lm(formula = focal_trait ~ treatment, data = data, weights = weights)) and its interaction with sex (R syntax: lm(formula = focal_trait ~ treatment + sex:treatment, data = data, weights = weights)). Visualized in Fig. 1A.

Trait	term	F	Estimate	SE	N treatment	N control	P
Leg length	Interaction by sex	0.16					0.688
	Boys	34.41	-0.50	0.08	159	3059	**0.000000005**
	Girls	28.57	-0.49	0.09	106	2548	**0.00000010**
Height	Interaction by sex	0.10					0.753
	Boys	24.51	-0.41	0.08	160	3067	**0.0000008**
	Girls	22.11	-0.43	0.09	106	2548	**0.000003**
Leg/torso ratio	Interaction by sex	0.07					0.798
	Boys	23.33	-0.42	0.09	159	3059	**0.000001**
	Girls	17.49	-0.39	0.09	106	2548	**0.00003**
Cranial volume	Interaction by sex	0.25					0.617
	Boys	17.64	-0.36	0.09	160	3066	**0.00003**
	Girls	18.13	-0.39	0.09	106	2548	**0.00002**
Hip width	Interaction by sex	1.12					0.289
	Boys	7.96	-0.23	0.08	159	3057	**0.005**
	Girls	15.63	-0.36	0.09	106	2541	**0.00008**
Face width	Interaction by sex	2.95					0.086
	Boys	16.78	-0.34	0.08	160	3064	**0.00004**
	Girls	5.98	-0.22	0.09	106	2547	**0.015**
Handgrip strenght	Interaction by sex	3.48					0.062
	Boys	18.45	-0.38	0.09	135	2582	**0.00002**
	Girls	2.46	-0.15	0.09	88	2120	0.117
Shoulder width	Interaction by sex	0.70					0.403
	Boys	4.19	-0.17	0.08	160	3060	**0.041**
	Girls	9.86	-0.29	0.09	106	2546	**0.002**
Sitting height	Interaction by sex	0.02					0.894
	Boys	3.99	-0.16	0.08	159	3059	**0.046**
	Girls	6.68	-0.24	0.09	106	2549	**0.010**
Weight	Interaction by sex	0.02					0.886
	Boys	5.14	-0.18	0.08	157	2802	**0.023**
	Girls	4.73	-0.18	0.08	104	2425	**0.030**
Thorax circumference	Interaction by sex	0.40					0.525
	Boys	0.12	0.03	0.08	160	3066	0.734
	Girls	0.43	-0.05	0.08	106	2548	0.512
Face roundness	Interaction by sex	4.96					**0.026**
	Boys	2.23	-0.13	0.08	160	3060	0.135
	Girls	1.44	0.12	0.10	106	2545	0.230
BMI	Interaction by sex	0.01					0.936
	Boys	1.07	0.09	0.08	157	2802	0.300
	Girls	0.78	0.07	0.08	104	2424	0.376
Shoulder/hip ratio	Interaction by sex	0.54					0.464
	Boys	0.20	0.04	0.08	160	3059	0.652
	Girls	2.15	0.13	0.09	106	2544	0.142

Table S1B. Effects of having mother dead on anthropometric traits of children (separately for boys and girls; R syntax: lm(formula = focal_trait ~ treatment, data = data, weights = weights)) and its interaction with sex (R syntax: lm(formula = focal_trait ~ treatment + sex:treatment, data = data, weights = weights)). Visualized in Fig. 1B.

Trait	term	F	Estimate	SE	N treatment	N control	P
Handgrip strenght	Interaction by sex	1.9					0.173
	Boys	6.6	-0.23	0.09	159	2763	**0.010**
	Girls	0.3	-0.05	0.08	174	3244	0.563
Leg length	Interaction by sex	3.0					0.082
	Boys	1.9	-0.11	0.08	180	3134	0.164
	Girls	0.6	-0.05	0.07	191	3563	0.457
Height	Interaction by sex	4.0					**0.046**
	Boys	2.9	-0.13	0.08	180	3142	0.086
	Girls	0.0	-0.01	0.07	191	3564	0.856
Leg/torso ratio	Interaction by sex	0.3					0.587
	Boys	0.1	-0.02	0.08	180	3134	0.799
	Girls	1.8	-0.10	0.07	191	3563	0.186
Weight	Interaction by sex	3.9					**0.047**
	Boys	4.8	-0.18	0.08	169	2930	**0.029**
	Girls	1.0	0.08	0.08	181	3389	0.308
Thorax circumference	Interaction by sex	4.4					**0.035**
	Boys	5.2	-0.18	0.08	180	3142	**0.023**
	Girls	1.2	0.08	0.07	191	3560	0.280
Shoulder width	Interaction by sex	6.3					**0.012**
	Boys	4.7	-0.17	0.08	179	3134	**0.031**
	Girls	1.1	0.08	0.08	191	3561	0.289
Cranial volume	Interaction by sex	3.9					**0.048**
	Boys	3.6	-0.15	0.08	180	3140	0.058
	Girls	0.6	0.06	0.08	191	3562	0.435
Hip width	Interaction by sex	2.4					0.119
	Boys	2.0	-0.11	0.08	180	3136	0.154
	Girls	0.1	0.03	0.07	191	3552	0.723
Sitting height	Interaction by sex	2.9					0.091
	Boys	2.4	-0.12	0.08	180	3136	0.121
	Girls	0.3	0.04	0.07	191	3563	0.585
Face width	Interaction by sex	9.5					**0.002**
	Boys	6.1	-0.19	0.08	180	3136	**0.013**
	Girls	4.2	0.15	0.07	191	3563	**0.041**
Shoulder/hip ratio	Interaction by sex	0.7					0.407
	Boys	0.9	-0.07	0.07	179	3134	0.348
	Girls	0.4	0.05	0.07	191	3557	0.508
BMI	Interaction by sex	2.9					0.087
	Boys	2.3	-0.13	0.08	169	2928	0.128
	Girls	2.5	0.12	0.08	181	3389	0.117
Face roundness	Interaction by sex	0.8					0.368
	Boys	0.5	-0.06	0.08	178	3133	0.468
	Girls	1.4	0.09	0.07	190	3555	0.232

Table S1C. Effects of having father dead on anthropometric traits of children (separately for boys and girls; R syntax: lm(formula = focal_trait ~ treatment, data = data, weights = weights)) and its interaction with sex (R syntax: lm(formula = focal_trait ~ treatment + sex:treatment, data = data, weights = weights)). Visualized in Fig. 1C.

Trait	term	F	Estimate	SE	N treatment	N control	P
Leg/torso ratio	Interaction by sex	0.15					0.696
	Boys	1.84	-0.05	0.03	999	6785	0.175
	Girls	3.50	-0.06	0.03	1401	8643	0.061
Leg length	Interaction by sex	1.58					0.209
	Boys	1.59	-0.04	0.03	999	6785	0.208
	Girls	0.45	-0.02	0.03	1401	8643	0.501
Height	Interaction by sex	3.69					0.055
	Boys	0.82	-0.03	0.03	999	6809	0.364
	Girls	0.03	0.01	0.03	1402	8648	0.864
Cranial volume	Interaction by sex	0.02					0.900
	Boys	0.03	-0.01	0.03	999	6805	0.869
	Girls	0.08	-0.01	0.03	1400	8642	0.779
Handgrip strenght	Interaction by sex	0.06					0.803
	Boys	0.04	0.01	0.04	935	5987	0.832
	Girls	0.00	0.00	0.03	1293	7477	0.949
Hip width	Interaction by sex	0.61					0.435
	Boys	0.02	0.00	0.04	999	6793	0.896
	Girls	0.66	0.02	0.03	1397	8628	0.415
Shoulder/hip ratio	Interaction by sex	1.03					0.311
	Boys	0.03	-0.01	0.03	998	6794	0.867
	Girls	1.17	0.03	0.03	1400	8635	0.280
Face roundness	Interaction by sex	2.33					0.127
	Boys	0.25	-0.02	0.03	994	6791	0.614
	Girls	2.81	0.05	0.03	1400	8623	0.093
Sitting height	Interaction by sex	4.71					**0.030**
	Boys	0.02	-0.01	0.04	999	6790	0.885
	Girls	1.70	0.04	0.03	1401	8644	0.192
Face width	Interaction by sex	2.50					0.114
	Boys	0.01	0.00	0.03	996	6803	0.915
	Girls	2.52	0.05	0.03	1401	8645	0.112
Shoulder width	Interaction by sex	7.02					**0.008**
	Boys	0.14	-0.01	0.03	998	6796	0.711
	Girls	4.61	0.06	0.03	1401	8640	**0.032**
Weight	Interaction by sex	2.60					0.107
	Boys	0.10	0.01	0.04	962	6438	0.755
	Girls	1.98	0.04	0.03	1353	8330	0.160
Thorax circumference	Interaction by sex	2.30					0.129
	Boys	0.00	0.00	0.04	999	6806	0.961
	Girls	4.21	0.06	0.03	1402	8645	**0.040**
BMI	Interaction by sex	0.51					0.473
	Boys	0.97	0.03	0.03	962	6434	0.325
	Girls	2.57	0.05	0.03	1353	8329	0.109

Table S1D. Effects of having father divorced on anthropometric traits of children (separately for boys and girls; R syntax: lm(formula = focal_trait ~ treatment, data = data, weights = weights)) and its interaction with sex (R syntax: lm(formula = focal_trait ~ treatment + sex:treatment, data = data, weights = weights)). Visualized in Fig. 1D.

Trait	term	F	Estimate	SE	N treatment	N control	P
Cranial volume	Interaction by sex	0.94					0.332
	Boys	2.98	-0.11	0.06	254	2991	0.084
	Girls	0.15	-0.02	0.05	588	5785	0.698
Leg/torso ratio	Interaction by sex	3.08					0.079
	Boys	2.67	-0.10	0.06	254	2985	0.102
	Girls	0.02	-0.01	0.04	588	5786	0.882
Hip width	Interaction by sex	1.47					0.225
	Boys	1.55	-0.08	0.06	252	2983	0.213
	Girls	0.09	-0.01	0.04	588	5774	0.759
Leg length	Interaction by sex	4.34					**0.037**
	Boys	1.30	-0.07	0.06	254	2985	0.255
	Girls	0.06	0.01	0.04	588	5786	0.808
Handgrip strenght	Interaction by sex	0.02					0.893
	Boys	0.45	-0.04	0.07	193	2443	0.501
	Girls	0.03	-0.01	0.05	452	4623	0.869
Face roundness	Interaction by sex	0.03					0.873
	Boys	0.16	-0.03	0.06	254	2975	0.688
	Girls	0.09	-0.01	0.04	588	5771	0.762
BMI	Interaction by sex	0.20					0.653
	Boys	0.01	-0.01	0.06	250	2733	0.922
	Girls	0.26	-0.02	0.05	579	5598	0.607
Weight	Interaction by sex	0.21					0.646
	Boys	0.14	-0.02	0.06	250	2735	0.710
	Girls	0.00	0.00	0.05	579	5598	0.958
Face width	Interaction by sex	0.43					0.511
	Boys	0.38	-0.04	0.06	254	2988	0.539
	Girls	0.13	0.02	0.05	588	5787	0.719
Thorax circumference	Interaction by sex	0.15					0.696
	Boys	0.00	0.00	0.06	253	2991	0.950
	Girls	0.04	-0.01	0.05	587	5788	0.851
Shoulder width	Interaction by sex	0.07					0.793
	Boys	0.00	0.00	0.06	253	2989	0.997
	Girls	0.03	-0.01	0.04	587	5786	0.856
Height	Interaction by sex	2.75					0.097
	Boys	0.15	-0.02	0.06	254	2993	0.697
	Girls	0.25	0.02	0.04	588	5790	0.616
Sitting height	Interaction by sex	0.53					0.466
	Boys	0.42	0.04	0.06	254	2988	0.517
	Girls	0.54	0.03	0.04	588	5786	0.462
Shoulder/hip ratio	Interaction by sex	3.57					0.059
	Boys	3.60	0.13	0.07	254	2986	0.058
	Girls	0.08	0.01	0.04	587	5779	0.783

Table S1E. Effects of having father divorced vs dead on anthropometric traits of children (separately for boys and girls; R syntax: lm(formula = focal_trait ~ treatment, data = data, weights = weights)) and its interaction with sex (R syntax: lm(formula = focal_trait ~ treatment + sex:treatment, data = data, weights = weights)). Visualized in Fig. 1E.

Trait	term	F	Estimate	SE	N treatment	N control	P
Face roundness	Interaction by sex	0.04					0.844
	Boys	0.08	-0.03	0.09	182	317	0.773
	Girls	0.87	-0.06	0.06	410	665	0.351
Handgrip strenght	Interaction by sex	0.43					0.512
	Boys	0.60	0.07	0.09	154	298	0.440
	Girls	0.49	-0.05	0.06	333	609	0.482
BMI	Interaction by sex	0.31					0.575
	Boys	1.94	0.13	0.09	179	297	0.165
	Girls	0.80	-0.06	0.07	404	639	0.370
Thorax circumference	Interaction by sex	0.30					0.587
	Boys	2.75	0.15	0.09	181	319	0.098
	Girls	1.29	-0.08	0.07	409	665	0.257
Weight	Interaction by sex	0.31					0.580
	Boys	1.45	0.12	0.10	179	297	0.229
	Girls	0.32	-0.04	0.07	404	639	0.571
Sitting height	Interaction by sex	0.82					0.365
	Boys	2.82	0.15	0.09	182	319	0.094
	Girls	1.29	-0.07	0.06	410	664	0.257
Hip width	Interaction by sex	1.07					0.300
	Boys	3.29	0.16	0.09	180	319	0.070
	Girls	1.10	-0.07	0.06	410	664	0.294
Leg/torso ratio	Interaction by sex	1.62					0.203
	Boys	0.12	-0.03	0.09	182	319	0.732
	Girls	4.91	0.14	0.06	410	664	**0.027**
Leg length	Interaction by sex	3.61					0.058
	Boys	0.61	0.07	0.09	182	319	0.435
	Girls	0.89	0.06	0.06	410	664	0.347
Height	Interaction by sex	2.70					0.101
	Boys	1.95	0.12	0.09	182	319	0.163
	Girls	0.00	0.00	0.06	410	665	0.944
Shoulder/hip ratio	Interaction by sex	5.05					**0.025**
	Boys	1.35	0.12	0.10	182	318	0.245
	Girls	0.12	0.02	0.06	409	665	0.729
Cranial volume	Interaction by sex	1.49					0.222
	Boys	0.09	0.03	0.09	182	319	0.769
	Girls	3.60	0.12	0.06	410	664	0.058
Shoulder width	Interaction by sex	0.04					0.838
	Boys	5.42	0.21	0.09	182	318	**0.020**
	Girls	0.91	-0.06	0.06	409	665	0.341
Face width	Interaction by sex	2.40					0.121
	Boys	1.08	0.10	0.09	182	317	0.299
	Girls	2.21	0.09	0.06	410	665	0.138

Table S2. Basic demographic parameters for samples children from different types of disrupted families who were matched against samples children from-biparental families (columns 2 – 6) or vs a sample of children whose fathers were dead (column 6). YOB is year of birth, age is given in years

Trait	Orphanages	Mother dead	Father Dead	Father divorced	Father divorced vs dead
N	266	371	2401	842	591
Boys: girls	160:106	180:191	999:1402	254:588	182:410
YOB min	1936	1936	1936	1938	1938
YOB median	1947	1947	1945	1952	1951
YOB mean (SD)	1946.7 (3.7)	1946.9 (4.6)	1946.0 (4.6)	1951.5 (4.7)	1950.6 (4.7)
YOB max	1955	1961	1962	1962	1962
Age min	7.0	6.9	6.6	6.5	6.9
Age median	11.8	13.9	14.5	12.2	12.6
Age mean	12.2 (3.0)	13.6 (3.0)	14.1 (2.8)	12.2 (3.0)	12.5
Age max	19.8	20.6	20.6	18.8	18.5
Rural: Urban	134:32	167:204	926:1475	246:596	382:602
SEP: manual workers		237	1817	483	788
SEP: skilled workers		69	242	157	73
SEP: non-manual		65	342	202	123

Table S3A. Effects of orphan status on anthropometric traits of children (separately for boys and girls; R syntax same as S1. Control and treatment groups are matched within six months of age. Visualized in Fig. S2A.

Trait	term	F	Estimate	SE	N treatment	N control	P
Leg length	Interaction by sex	0.16					0.691
	Boys	33.63	-0.50	0.09	159	2162	**0.000**
	Girls	25.59	-0.48	0.09	106	1775	**0.000**
Height	Interaction by sex	0.10					0.755
	Boys	23.01	-0.40	0.08	160	2169	**0.000**
	Girls	20.10	-0.42	0.09	106	1775	**0.000**
Leg/torso ratio	Interaction by sex	0.06					0.800
	Boys	24.45	-0.43	0.09	159	2162	**0.000**
	Girls	15.63	-0.38	0.10	106	1775	**0.000**
Cranial volume	Interaction by sex	0.25					0.619
	Boys	15.75	-0.34	0.09	160	2168	**0.000**
	Girls	18.72	-0.41	0.09	106	1776	**0.000**
Hip width	Interaction by sex	1.12					0.291
	Boys	8.03	-0.24	0.08	159	2163	**0.005**
	Girls	15.31	-0.36	0.09	106	1771	**0.000**
Face width	Interaction by sex	3.02					0.082
	Boys	17.55	-0.34	0.08	160	2168	**0.000**
	Girls	7.64	-0.25	0.09	106	1774	**0.006**
Handgrip strength	Interaction by sex	3.56					0.059
	Boys	19.51	-0.38	0.09	135	1882	**0.000**
	Girls	2.37	-0.14	0.09	88	1462	0.124
Shoulder width	Interaction by sex	0.69					0.406
	Boys	4.07	-0.17	0.08	160	2163	**0.044**
	Girls	9.92	-0.29	0.09	106	1774	**0.002**
Sitting height	Interaction by sex	0.02					0.895
	Boys	3.20	-0.15	0.08	159	2162	0.074
	Girls	6.34	-0.23	0.09	106	1776	**0.012**
Weight	Interaction by sex	0.02					0.888
	Boys	4.44	-0.17	0.08	157	2012	**0.035**
	Girls	4.44	-0.18	0.09	104	1686	**0.035**
Face roundness	Interaction by sex	5.00					**0.025**
	Boys	2.80	-0.14	0.08	160	2165	0.094
	Girls	0.69	0.08	0.10	106	1772	0.405
Thorax circumference	Interaction by sex	0.39					0.534
	Boys	0.11	0.03	0.08	160	2168	0.743
	Girls	0.39	-0.05	0.08	106	1776	0.533
BMI	Interaction by sex	0.01					0.936
	Boys	1.20	0.09	0.08	157	2012	0.273
	Girls	0.61	0.07	0.09	104	1685	0.437
Shoulder/hip ratio	Interaction by sex	0.56					0.455
	Boys	0.24	0.04	0.08	160	2164	0.624
	Girls	2.18	0.13	0.09	106	1773	0.140

Table S3B. Effects of orphan status on anthropometric traits of children (separately for boys and girls; R syntax same as S1. Control and treatment groups are matched within six months of age. Visualized in Fig. 2B.

Trait	term	F	Estimate	SE	N treatment	N control	P
Handgrip strength	Interaction by sex	1.75					0.186
	Boys	4.37	-0.19	0.09	155	1745	**0.037**
	Girls	0.02	-0.01	0.08	170	1979	0.883
Leg length	Interaction by sex	3.07					0.080
	Boys	0.71	-0.07	0.08	176	1960	0.399
	Girls	0.26	-0.04	0.08	187	2152	0.614
Leg/torso ratio	Interaction by sex	0.23					0.633
	Boys	0.01	-0.01	0.08	176	1960	0.907
	Girls	1.49	-0.09	0.08	187	2152	0.222
Height	Interaction by sex	4.33					**0.038**
	Boys	1.22	-0.09	0.08	176	1964	0.269
	Girls	0.00	0.00	0.08	187	2153	0.953
Cranial volume	Interaction by sex	3.37					0.066
	Boys	2.76	-0.13	0.08	176	1962	0.097
	Girls	0.58	0.06	0.08	187	2152	0.445
Sitting height	Interaction by sex	3.46					0.063
	Boys	1.18	-0.09	0.08	176	1961	0.277
	Girls	0.53	0.06	0.08	187	2152	0.466
Shoulder width	Interaction by sex	6.59					**0.010**
	Boys	2.72	-0.13	0.08	175	1960	0.100
	Girls	1.63	0.10	0.08	187	2151	0.202
Hip width	Interaction by sex	2.49					0.115
	Boys	0.53	-0.06	0.08	176	1957	0.465
	Girls	0.20	0.03	0.08	187	2144	0.656
Shoulder/hip ratio	Interaction by sex	0.72					0.396
	Boys	1.24	-0.08	0.07	175	1959	0.265
	Girls	0.62	0.06	0.08	187	2148	0.429
Thorax circumference	Interaction by sex	4.26					**0.039**
	Boys	2.38	-0.12	0.08	176	1963	0.123
	Girls	2.03	0.11	0.08	187	2152	0.154
Weight	Interaction by sex	4.29					**0.038**
	Boys	2.35	-0.13	0.08	166	1827	0.125
	Girls	2.54	0.13	0.08	177	2036	0.111
Face roundness	Interaction by sex	0.81					0.369
	Boys	1.00	-0.08	0.08	174	1959	0.317
	Girls	1.69	0.10	0.08	186	2146	0.194
Face width	Interaction by sex	8.07					**0.005**
	Boys	3.51	-0.15	0.08	176	1961	0.061
	Girls	4.93	0.17	0.08	187	2152	**0.026**
BMI	Interaction by sex	3.16					0.076
	Boys	1.51	-0.10	0.08	166	1826	0.219
	Girls	4.42	0.17	0.08	177	2036	**0.036**

Table S3C. Effects of orphan status on anthropometric traits of children (separately for boys and girls; R syntax same as S1. Control and treatment groups are matched within six months of age. Fig. S2C.

Trait	term	F	Estimate	SE	N treatment	N control	P
Leg/torso ratio	Interaction by sex	0.24					0.624
	Boys	1.80	-0.05	0.04	983	5291	0.180
	Girls	4.33	-0.06	0.03	1389	6916	**0.037**
Leg length	Interaction by sex	1.67					0.197
	Boys	0.96	-0.04	0.04	983	5291	0.327
	Girls	0.36	-0.02	0.03	1389	6916	0.551
Cranial volume	Interaction by sex	0.02					0.899
	Boys	0.26	-0.02	0.03	983	5306	0.608
	Girls	0.00	0.00	0.03	1388	6917	0.979
Height	Interaction by sex	4.03					**0.045**
	Boys	0.31	-0.02	0.04	983	5310	0.576
	Girls	0.14	0.01	0.03	1390	6921	0.705
Hip width	Interaction by sex	0.73					0.394
	Boys	0.03	-0.01	0.04	983	5295	0.862
	Girls	0.66	0.02	0.03	1385	6903	0.418
Face roundness	Interaction by sex	2.09					0.148
	Boys	0.25	-0.02	0.03	978	5298	0.619
	Girls	1.42	0.04	0.03	1388	6903	0.233
Face width	Interaction by sex	2.49					0.115
	Boys	0.07	-0.01	0.03	980	5305	0.788
	Girls	2.16	0.04	0.03	1389	6920	0.141
Handgrip strength	Interaction by sex	0.12					0.728
	Boys	0.48	0.03	0.04	922	4832	0.488
	Girls	0.08	0.01	0.03	1282	6098	0.781
Shoulder/hip ratio	Interaction by sex	0.98					0.322
	Boys	0.01	0.00	0.03	982	5295	0.916
	Girls	1.45	0.04	0.03	1388	6909	0.229
Sitting height	Interaction by sex	5.31					**0.021**
	Boys	0.03	0.01	0.04	983	5295	0.870
	Girls	2.70	0.05	0.03	1389	6917	0.100
Thorax circumference	Interaction by sex	2.51					0.113
	Boys	0.00	0.00	0.04	983	5307	0.980
	Girls	4.04	0.06	0.03	1390	6918	**0.044**
Shoulder width	Interaction by sex	7.30					**0.007**
	Boys	0.05	-0.01	0.04	982	5297	0.821
	Girls	5.50	0.07	0.03	1389	6914	**0.019**
Weight	Interaction by sex	2.60					0.107
	Boys	0.25	0.02	0.04	946	4986	0.614
	Girls	2.03	0.05	0.03	1341	6670	0.154
BMI	Interaction by sex	0.43					0.514
	Boys	0.82	0.03	0.04	946	4983	0.365
	Girls	2.26	0.05	0.03	1341	6669	0.132

Table S3D. Effects of orphan status on anthropometric traits of children (separately for boys and girls; R syntax same as S1. Control and treatment groups are matched within six months of age. Fig. S2D.

Trait	term	F	Estimate	SE	N treatment	N control	P
Leg/torso ratio	Interaction by sex	3.39					0.066
	Boys	2.80	-0.10	0.06	253	2101	0.094
	Girls	0.00	0.00	0.05	583	3997	0.947
Cranial volume	Interaction by sex	1.10					0.295
	Boys	2.61	-0.10	0.06	253	2103	0.107
	Girls	0.00	0.00	0.05	583	3994	0.983
Hip width	Interaction by sex	1.52					0.217
	Boys	1.02	-0.06	0.06	251	2100	0.312
	Girls	0.48	-0.03	0.05	583	3986	0.487
Face roundness	Interaction by sex	0.02					0.884
	Boys	0.35	-0.04	0.07	253	2097	0.552
	Girls	0.17	-0.02	0.05	583	3987	0.678
Leg length	Interaction by sex	4.78					**0.029**
	Boys	0.77	-0.05	0.06	253	2101	0.381
	Girls	0.00	0.00	0.05	583	3997	0.981
Face width	Interaction by sex	0.54					0.461
	Boys	0.40	-0.04	0.06	253	2102	0.525
	Girls	0.25	0.02	0.05	583	3997	0.615
BMI	Interaction by sex	0.21					0.643
	Boys	0.00	0.00	0.07	249	1910	0.954
	Girls	0.06	-0.01	0.05	574	3866	0.811
Weight	Interaction by sex	0.23					0.629
	Boys	0.00	0.00	0.06	249	1912	0.958
	Girls	0.00	0.00	0.05	574	3866	0.970
Handgrip strength	Interaction by sex	0.04					0.845
	Boys	0.01	-0.01	0.07	192	1773	0.922
	Girls	0.00	0.00	0.05	449	3224	0.950
Height	Interaction by sex	3.11					0.078
	Boys	0.00	0.00	0.06	253	2105	0.988
	Girls	0.02	0.01	0.04	583	3999	0.885
Thorax circumference	Interaction by sex	0.15					0.702
	Boys	0.12	0.02	0.06	252	2102	0.725
	Girls	0.08	-0.01	0.05	582	3999	0.783
Shoulder width	Interaction by sex	0.05					0.819
	Boys	0.07	0.02	0.06	252	2104	0.798
	Girls	0.00	0.00	0.05	582	3997	0.989
Sitting height	Interaction by sex	0.63					0.427
	Boys	1.17	0.06	0.06	253	2103	0.280
	Girls	0.09	0.01	0.04	583	3997	0.769
Shoulder/hip ratio	Interaction by sex	3.48					0.062
	Boys	3.56	0.13	0.07	253	2102	0.059
	Girls	0.76	0.04	0.04	582	3990	0.383

Table S3E. Effects of orphan status on anthropometric traits of children (separately for boys and girls; R syntax same as S1. Control and treatment groups are matched within six months of age. Fig. S2E.

Trait	term	F	Estimate	SE	N treatment	N control	P
Face roundness	Interaction by sex	0.30					0.585
	Boys	1.14	-0.11	0.11	147	219	0.287
	Girls	3.04	-0.12	0.07	314	459	0.082
Handgrip strength	Interaction by sex	0.04					0.836
	Boys	0.05	0.02	0.11	131	211	0.827
	Girls	1.22	-0.09	0.08	254	420	0.269
Face width	Interaction by sex	2.03					0.155
	Boys	0.21	-0.05	0.11	147	219	0.648
	Girls	0.00	0.00	0.07	314	459	0.969
BMI	Interaction by sex	0.05					0.829
	Boys	0.39	0.07	0.11	145	203	0.533
	Girls	0.61	-0.06	0.08	308	441	0.434
Cranial volume	Interaction by sex	2.20					0.138
	Boys	0.00	0.00	0.11	147	221	0.996
	Girls	0.47	0.05	0.08	314	459	0.495
Hip width	Interaction by sex	0.94					0.332
	Boys	2.95	0.17	0.10	145	221	0.086
	Girls	0.88	-0.07	0.07	314	458	0.347
Shoulder width	Interaction by sex	0.20					0.655
	Boys	3.52	0.19	0.10	147	220	0.061
	Girls	1.53	-0.09	0.07	313	459	0.217
Shoulder/hip ratio	Interaction by sex	2.41					0.121
	Boys	0.78	0.10	0.12	147	220	0.378
	Girls	0.01	0.01	0.07	313	459	0.921
Sitting height	Interaction by sex	2.24					0.135
	Boys	2.55	0.17	0.10	147	221	0.111
	Girls	0.36	-0.04	0.07	314	458	0.550
Weight	Interaction by sex	0.77					0.382
	Boys	2.41	0.16	0.11	145	203	0.121
	Girls	0.13	-0.03	0.08	308	441	0.714
Thorax circumference	Interaction by sex	0.97					0.325
	Boys	4.07	0.20	0.10	146	221	**0.044**
	Girls	0.10	-0.03	0.08	314	459	0.753
Leg/torso ratio	Interaction by sex	0.01					0.905
	Boys	0.66	0.09	0.11	147	221	0.419
	Girls	2.31	0.11	0.07	314	458	0.129
Height	Interaction by sex	2.04					0.154
	Boys	4.54	0.22	0.10	147	221	**0.034**
	Girls	0.05	0.02	0.07	314	459	0.818
Leg length	Interaction by sex	1.12					0.291
	Boys	4.04	0.20	0.10	147	221	**0.045**
	Girls	0.54	0.05	0.07	314	458	0.461

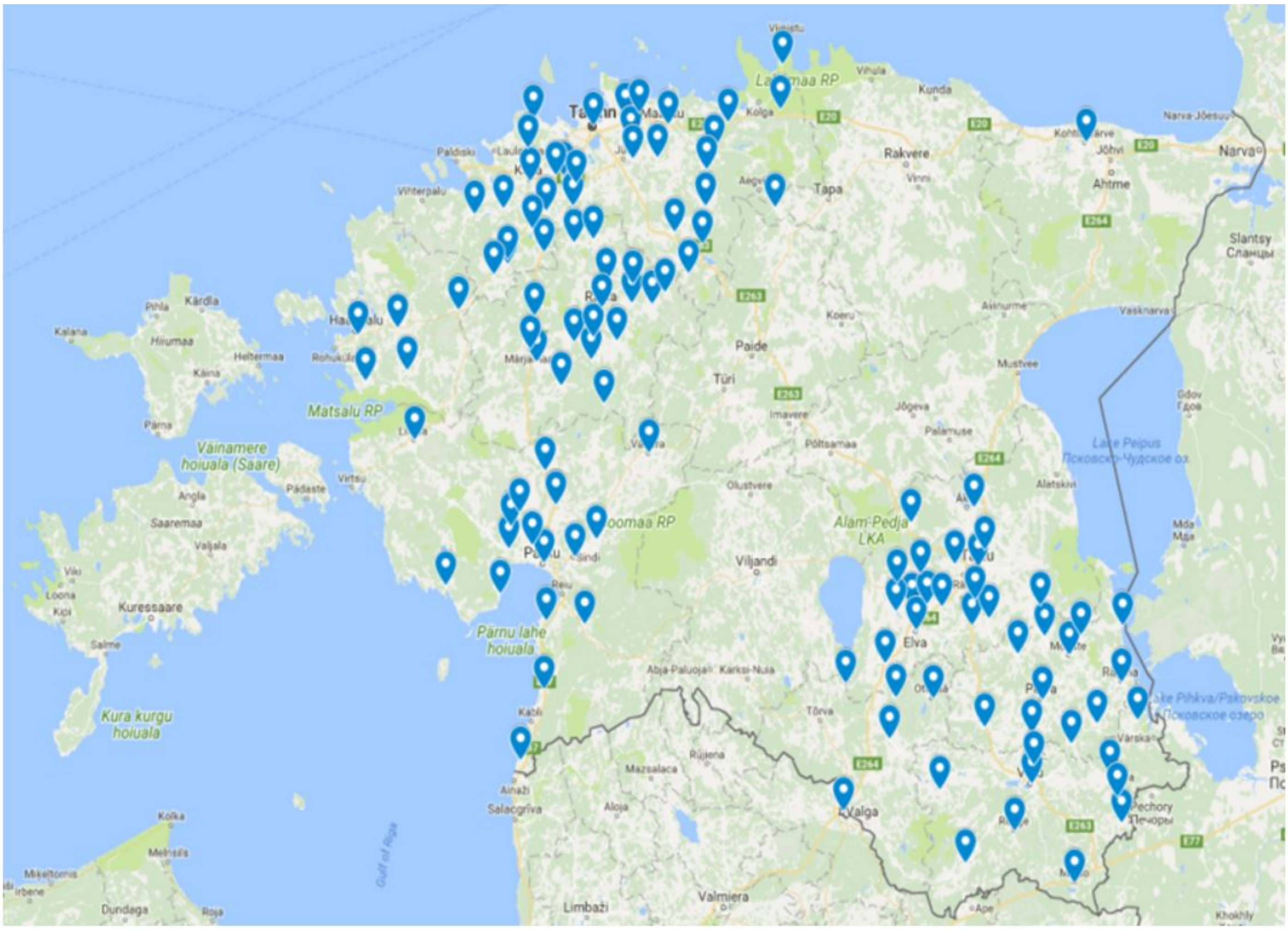

Fig. S1. Location of schools where Juhan Aul collected anthropometric measures from 1956 to 1968

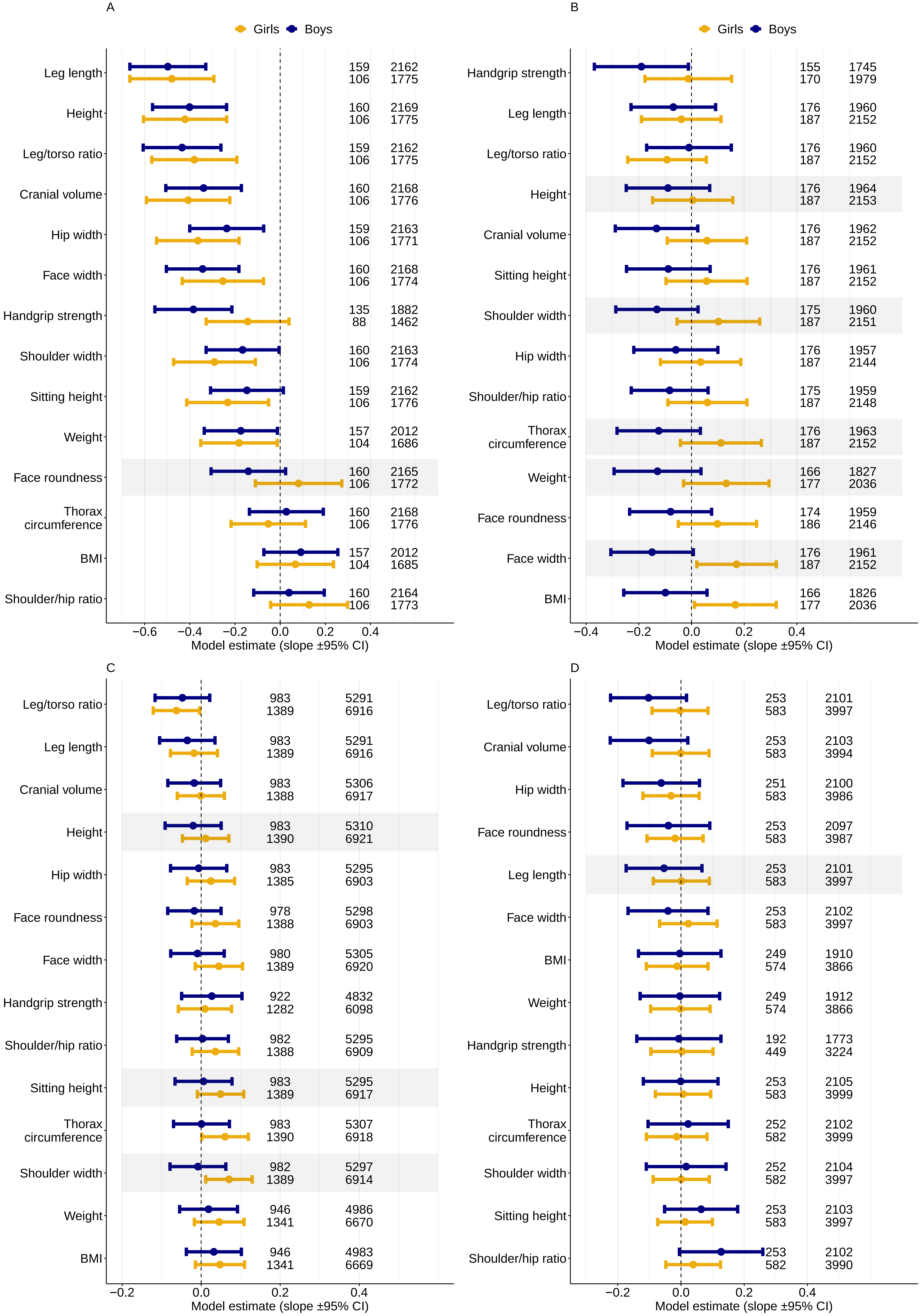
A
Girls Boys
Leg length 159 106 2162 1775
Height 160 106 2169 1775
Leg/torso ratio 159 106 2162 1775
Cranial volume 160 106 2168 1776
Hip width 159 106 2163 1771
Face width 160 106 2168 1774
Handgrip strength 135 88 1882 1462
Shoulder width 160 106 2163 1774
Sitting height 159 106 2162 1776
Weight 157 104 2012 1686
Face roundness 160 106 2165 1772
Thorax circumference 160 106 2168 1776
BMI 157 104 2012 1685
Shoulder/hip ratio 160 106 2164 1773
−0.6 −0.4 −0.2 0.0 0.2 0.4
Model estimate (slope ±95% CI)
B
Girls Boys
Handgrip strength 155 170 1745 1979
Leg length 176 187 1960 2152
Leg/torso ratio 176 187 1960 2152
Height 176 187 1964 2153
Cranial volume 176 187 1962 2152
Sitting height 176 187 1961 2152
Shoulder width 175 187 1960 2151
Hip width 176 187 1957 2144
Shoulder/hip ratio 175 187 1959 2148
Thorax circumference 176 187 1963 2152
Weight 166 177 1827 2036
Face roundness 174 186 1959 2146
Face width 176 187 1961 2152
BMI 166 177 1826 2036
−0.4 −0.2 0.0 0.2 0.4
Model estimate (slope ±95% CI)
C
Leg/torso ratio 983 1389 5291 6916
Leg length 983 1389 5291 6916
Cranial volume 983 1388 5306 6917
Height 983 1390 5310 6921
Hip width 983 1385 5295 6903
Face roundness 978 1388 5298 6903
Face width 980 1389 5305 6920
Handgrip strength 922 1282 4832 6098
Shoulder/hip ratio 982 1388 5295 6909
Sitting height 983 1389 5295 6917
Thorax circumference 983 1390 5307 6918
Shoulder width 982 1389 5297 6914
Weight 946 1341 4986 6670
BMI 946 1341 4983 6669
−0.2 0.0 0.2 0.4
Model estimate (slope ±95% CI)
D
Leg/torso ratio 253 583 2101 3997
Cranial volume 253 583 2103 3994
Hip width 251 583 2100 3986
Face roundness 253 583 2097 3987
Leg length 253 583 2101 3997
Face width 253 583 2102 3997
BMI 249 574 1910 3866
Weight 249 574 1912 3866
Handgrip strength 192 449 1773 3224
Height 253 583 2105 3999
Thorax circumference 252 582 2102 3999
Shoulder width 252 582 2104 3997
Sitting height 253 583 2103 3997
Shoulder/hip ratio 253 582 2102 3990
−0.2 0.0 0.2 0.4
Model estimate (slope ±95% CI)

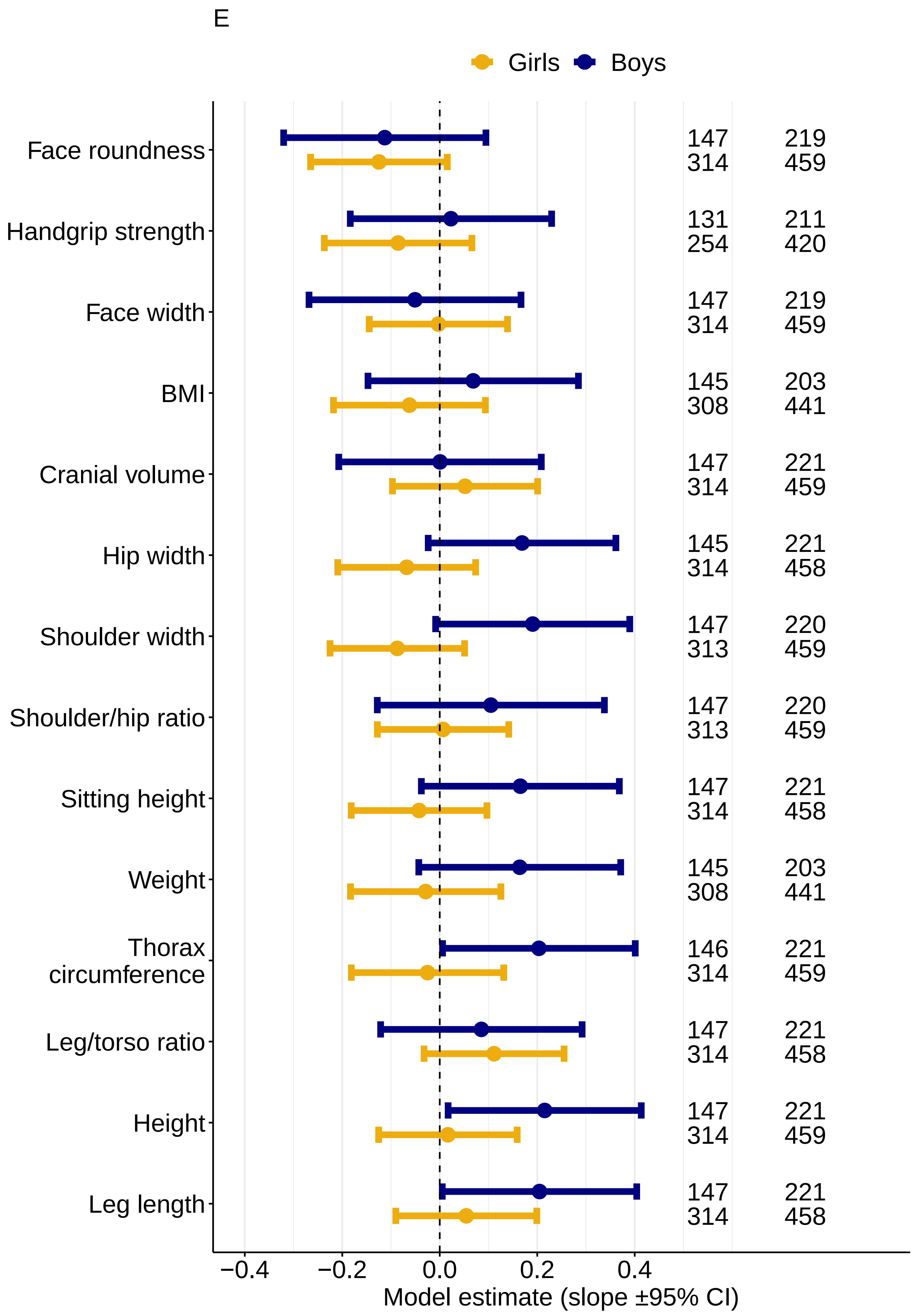

Fig. S2. Comparison of anthropometric traits in disrupted vs bi-parental families. Control and treatment groups are matched within the 6 months of age. Regression coefficients equal the differences between mean trait values of children in control and treatment groups in SD units in linear models. Sample sizes for treatment and control groups are indicated at right. Shaded areas denote significant trait*treatment interaction terms. F-statistics and p-values are given in Table S1 in ESM1. **A**: children from orphanages vs controls. **B**: children with dead mothers vs controls. **C**: children with dead fathers vs controls. **D**: children with divorced fathers vs controls. **E**: children with divorced fathers vs children with divorced fathers.

Find Matching Observations in Aul's data

Markus Valge, Richard Meitern and Peeter Hõrak

25/05/2021

Introduction

The aim of this document is to find matching controls for orphans and other subgroups in the the full data ("./data/aufam.rds"). This is need to perform a **Retrospective cohort study with multiple controls**.

Load functions

```
library("MatchIt")
```

```
#' Prepare Original Data for Matching
#'
#'This function removes rows with missing values from the input data
#' so it could be used for matching according to the formula.
#'
#' @param df the data on witch the matching is performed
#' @param formula the formula used for matching
#' @param control a column name in data that contains (1,0) values  defining
#' whether this row belongs to control group or not
#'
#' @return a data frame with the NA containing rows removed
#' @export
#'
prepareMathItDF <- function(df, formula, control = "both_parents"){
  f <- as.formula(formula)
  vars <- all.vars(f)
  treat <- vars[1]
  covariates <- vars[2:length(vars)]
  df[treat] <- ifelse(df[treat] == 1, 1, NA)
  df[treat] <- ifelse(df[[control]], 0, df[[treat]])
  cc <- complete.cases(df[c(treat,covariates)])
  df[cc,]
}
```

Load data

```
aufam <- readRDS("./data/aufam.rds")
```

```
#columns needed for the matching procedure
matchCols <- c(
          "age_yrs",
          "sex",
          "YOB",
          "Rural",
          "stepfam",
          "max_SEP_3",
          "both_parents",
          "orphan",
          "fa_dead_before",
          "fa_divorced",
          "mo_dead_before"
          )

#vector of columns needed for the subsequent statistical analyses in the format:
#"column_name" = "name in figures"
statCols <- c(
  "weight_rsz" = "Weight",
  "HGS_rzs" = "Handgrip strength",
  "height_rzs" = "Height",
  "cran_rzs" = "Cranial volume",
  "thorax_circ_rsz" = "Thorax circumference",
  "sho_to_hip_rsz" = "Shoulder/hip ratio",
  "sit_height_rsz"= "Sitting height",
  "sho_width_rsz"= "Shoulder width",
  "BMI_rsz"= "BMI",
  "face_width_rsz"= "Face width",
  "leg_rsz"= "Leg length",
  "hip_width_rsz"= "Hip width",
  "face_roundness_rsz"= "Face roundness",
  "leg_to_torso_rsz"= "Leg/torso ratio"
  )
```

The approximate age of children was calculated by rounding the exact age in years to the nearest integer. For more precise matching by date of birth a year of birth was divided into half.

```
aufam$age_aprox <- round(aufam$age_yrs)
aufam$yearPart <- ifelse(lubridate::month(aufam$birth_date) < 7,
                              "I",  "II")
aufam$YOB <- lubridate::year(aufam$birth_date)
```

Preform exact matching against children with both parents

To perform more precise matching the term *yearPart* was included in the matching formulas below. The results using data from these matchers are presented in the supplementary tables and figures. To exactly reproduce the results displayed in the main text the term *yearPart* should be removed from the *matchForm*'s below.

```
# it can be changed to any other directory to save the matching data
outputDir <- "./data/halfYearMatch/"
```

Orphans

```
#exlucde children that are orphans but live in a stepfamily
#ie include only those that live in an orphanage
orphanage_df <- aufam[(aufam$stepfam == 0 & aufam$orphan) | !aufam$orphan, ]
```

```
matchForm <- orphan ~  YOB + sex + Rural + age_aprox + yearPart

orphanageMatch <- matchit(matchForm,
                        data=prepareMathItDF(orphanage_df, matchForm),
                        method = "exact")


# save the data frame for statistical analysis to a data subfolder
fname <- "orphanageMatch.rds"
saveRDS(match.data(orphanageMatch), paste0(outputDir,fname))
```

Children with dead fathers

```
matchForm <- fa_dead_before ~ YOB + sex + Rural + max_SEP_3 + age_aprox + yearPart

f_dead_beforeMatch <- matchit(matchForm,
                        data=prepareMathItDF(aufam, matchForm),
                        method = "exact")

# save the data frame for statistical analysis to a data subfolder
fname <- "fa_dead_beforeMatch.rds"
saveRDS(match.data(f_dead_beforeMatch), paste0(outputDir,fname))
```

Children with divorced fathers

```
matchForm <- fa_divorced ~ YOB + sex + Rural + max_SEP_3 +  age_aprox + yearPart

f_divorcedMatch <- matchit(matchForm,
                        data=prepareMathItDF(aufam, matchForm),
                        method = "exact")

#save the data frame for statistical analysis to a data subfolder
fname <- "f_divorcedMatch.rds"
saveRDS(match.data(f_divorcedMatch), paste0(outputDir,fname))
```

Children with dead mothers

```
matchForm <-  mo_dead_before ~ YOB + sex + Rural + max_SEP_3 + age_aprox + yearPart

mo_dead_beforeMatch <- matchit(matchForm,
                        data=prepareMathItDF(aufam, matchForm),
```

```
                        method = "exact")

#save the data frame for statistical analysis to a data subfolder
fname <- "mo_dead_beforeMatch.rds"
saveRDS(match.data(mo_dead_beforeMatch), paste0(outputDir,fname))
```

Preform exact matching against children with dead fathers

Children with divorced fathers

```
matchForm <- fa_divorced ~  YOB + sex + Rural + max_SEP_3 +  age_aprox + yearPart
fdiv_fdMatch <- matchit(matchForm,
                        data=prepareMathItDF(aufam, matchForm, "fa_dead_before"),
                        method = "exact")
# save the data frame for statistical analysis to a data subfolder
fname <- "fdiv_fdMatch.rds"
saveRDS(match.data(fdiv_fdMatch), paste0(outputDir,fname))
```

```
#END
```
